# Relationships between infection with *Plasmodium falciparum* during pregnancy, measures of placental malaria, and adverse birth outcomes

**DOI:** 10.1186/s12936-017-2040-4

**Published:** 2017-10-05

**Authors:** James Kapisi, Abel Kakuru, Prasanna Jagannathan, Mary K. Muhindo, Paul Natureeba, Patricia Awori, Miriam Nakalembe, Richard Ssekitoleko, Peter Olwoch, John Ategeka, Patience Nayebare, Tamara D. Clark, Gabrielle Rizzuto, Atis Muehlenbachs, Diane V. Havlir, Moses R. Kamya, Grant Dorsey, Stephanie L. Gaw

**Affiliations:** 1 School of Medicine, Makerere University College of Health Sciences, Kampala, Uganda; 2 Infectious Diseases Research Collaboration, Kampala, Uganda; 3 Department of Medicine, University of California, San Francisco, CA USA; 4 Department of Obstetrics and Gynecology, Makerere University College of Health Sciences, Kampala, Uganda; 5 Department of Pathology, University of California, San Francisco, CA USA; 6 Division of High-Consequence Pathogens and Pathology, Centers for Disease Control and Prevention, Atlanta, GA USA; 7 Division of Maternal-Fetal Medicine, Department of Obstetrics, Gynecology and Reproductive Sciences, University of California, San Francisco, CA USA; 8 Division of Infectious Diseases and Geographical Medicine, Stanford University, Stanford, CA USA

**Keywords:** Malaria, Pregnancy, Placental malaria, Birth outcomes, Asymptomatic parasitaemia, IPTp, Low birth weight, Small for gestational age, Preterm birth, LAMP

## Abstract

**Background:**

Malaria in pregnancy has been associated with maternal morbidity, placental malaria, and adverse birth outcomes. However, data are limited on the relationships between longitudinal measures of malaria during pregnancy, measures of placental malaria, and birth outcomes.

**Methods:**

This is a nested observational study of data from a randomized controlled trial of intermittent preventive therapy during pregnancy among 282 participants with assessment of placental malaria and delivery outcomes. HIV-uninfected pregnant women were enrolled at 12–20 weeks of gestation. Symptomatic malaria during pregnancy was measured using passive surveillance and monthly detection of asymptomatic parasitaemia using loop-mediated isothermal amplification (LAMP). Placental malaria was defined as either the presence of parasites in placental blood by microscopy, detection of parasites in placental blood by LAMP, or histopathologic evidence of parasites or pigment. Adverse birth outcomes assessed included low birth weight (LBW), preterm birth (PTB), and small for gestational age (SGA) infants.

**Results:**

The 282 women were divided into three groups representing increasing malaria burden during pregnancy. Fifty-two (18.4%) had no episodes of symptomatic malaria or asymptomatic parasitaemia during the pregnancy, 157 (55.7%) had low malaria burden (0–1 episodes of symptomatic malaria and < 50% of samples LAMP+), and 73 (25.9%) had high malaria burden during pregnancy (≥ 2 episodes of symptomatic malaria or ≥ 50% of samples LAMP+). Women with high malaria burden had increased risks of placental malaria by blood microscopy and LAMP [aRR 14.2 (1.80–111.6) and 4.06 (1.73–9.51), respectively], compared to the other two groups combined. Compared with women with no malaria exposure during pregnancy, the risk of placental malaria by histopathology was higher among low and high burden groups [aRR = 3.27 (1.32–8.12) and aRR = 7.07 (2.84–17.6), respectively]. Detection of placental parasites by any method was significantly associated with PTB [aRR 5.64 (1.46–21.8)], and with a trend towards increased risk for LBW and SGA irrespective of the level of malaria burden during pregnancy.

**Conclusion:**

Higher malaria burden during pregnancy was associated with placental malaria and together with the detection of parasites in the placenta were associated with increased risk for adverse birth outcomes.

*Trial Registration* Current Controlled Trials Identifier NCT02163447

**Electronic supplementary material:**

The online version of this article (doi:10.1186/s12936-017-2040-4) contains supplementary material, which is available to authorized users.

## Background

Malaria in pregnancy remains a major public health problem in many parts of sub-Saharan Africa partly because the coverage of malaria control measures is still low. The proportion of the population sleeping under an ITN and protected by IRS was estimated to be 57% in 2015 and only 31% of eligible pregnant women received three or more doses of IPTp in the same year [1]. In 2015, an estimated 28 million pregnant women were at risk of malaria in this region [1], and several studies have shown that the median prevalence of placental malaria is 26–28% in all pregnant women [2, 3]. Placental malaria is associated with adverse birth outcomes such as low birth weight (LBW) and preterm birth (PTB) [4, 5]. Approximately 20% of all LBW deliveries in Africa are attributable to malaria in pregnancy, leading to 75,000–200,000 infant deaths annually [2, 6]. Furthermore, malaria in pregnancy is associated with approximately 36% of all preterm births in endemic regions [3].

As most available data are from cross-sectional studies at the time of delivery, there are limited longitudinal data on malaria during pregnancy, and the impact of gestational malaria burden on measures of placental malaria and birth outcomes. A prospective study in Benin showed an association between microscopic and submicroscopic infections (detected by PCR) measured early in pregnancy with and increased risk of LBW and PTB, respectively [7], but did not investigate the relationship between placental malaria and adverse birth outcomes. An observational study in Uganda reported that malaria in pregnancy (diagnosed by peripheral microscopy) was associated with LBW and PTB [8]; however, pregnant women often have low levels of parasitaemia that require more sensitive molecular methods for detection, such as loop-mediated isothermal amplification (LAMP) [9]. A Malawian study found an association between submicroscopic peripheral malaria diagnosed by qPCR and the prevalence of placental malaria, but no associations between submicroscopic infection and adverse birth outcomes [10].

The overall objective of this study was to fill the evidence gap on the relationships between longitudinal measures of malaria burden during pregnancy, placental malaria and birth outcomes. Factors associated with the frequency of symptomatic malaria and asymptomatic parasitaemia during pregnancy were examined, and the associations between malaria burden and measures of placental malaria and adverse birth outcomes assessed.

## Methods

### Study design, site and population

This was a cohort study that utilized data from a randomized controlled trial of IPTp in Tororo, Uganda. Tororo is a rural district in southeastern Uganda with an entomologic inoculation rate estimated at 310 infectious bites per person year in 2012 [11]. From June 2014 to October 2014, 300 pregnant women were enrolled into a three-arm, double-blinded, placebo-controlled trial of sulfadoxine-pyrimethamine (SP) given every 8 weeks versus dihydroartemisinin-piperaquine (DP) given every 8 weeks versus DP given every 4 weeks for IPTp. Details of the parent study have been described elsewhere [12]. Briefly, participants were HIV-negative pregnant women of at least 16 years of age, with an estimated gestational age of 12–20 weeks confirmed by ultrasound. For the present study, all 282 women from this cohort with placental histopathology and known birth outcomes were included.

### Study procedures and follow-up

At enrollment, pregnant women underwent a standardized history and physical exam including assessment of wealth status, gravidity, gestational age by ultrasound, and age. Each participant received a long-lasting insecticide-treated bed net (LLIN). Women received all their medical care at a designated study clinic that was open daily. Routine visits were conducted every 4 weeks, including collection of dried blood spots (DBS) for LAMP. Participants with positive LAMP results were not treated for malaria. Women were also encouraged to present to the clinic with any illness. Patients who presented with a documented fever (tympanic temperature ≥ 38.0 °C) or a history of fever in the prior 24 h had peripheral blood collected for a thick blood smear. If the blood smear was positive, the patient was diagnosed with symptomatic malaria and treated with artemether–lumefantrine. At delivery, a standardized assessment was completed including evaluation of infant birth weight and gestational age, and collection of specimens within 1 h of delivery, including placental tissue, placental blood smears, and DBS of placental blood. All women were encouraged to deliver at the hospital adjacent to the study clinic. Women delivering at home were visited by study staff as close to the time of delivery as possible for assessment and sample collection.

### Laboratory methods

DBS were tested for the presence of malaria parasites using LAMP as previously described [13, 14]. Formalin-fixed paraffin-embedded placental biopsies were processed in duplicate for histological evidence of placental malaria using a standardized case record form by two independent readers as previously described [12]. A third reader resolved any discrepant results. Blood smears were stained with 2% Giemsa and read by two trained laboratory technicians. Smears were considered negative if no asexual parasites were detected in 100 high-powered fields. A third reviewer settled any discrepant readings.

### Variables of interest

The following demographic data was collected: maternal age, possession of bed net at enrollment, wealth status, gravidity, gestational age, and IPTp treatment arm. Wealth status was categorized into lowest, middle, and highest tertiles designed as a composite variable using ownership of several household items and land. Gravidity was grouped as primigravidas (1st pregnancy) and multigravidas (≥ 2 pregnancies). Gestational age at enrollment was confirmed by ultrasound measurements by fetal biometry at less than 20 weeks’ gestation. IPTp treatment arm was categorized as SP given every 8 weeks, DP given every 8 weeks and DP given every 4 weeks.

Malaria in pregnancy was defined as both symptomatic malaria and asymptomatic parasitaemia. Symptomatic malaria was measured using passive surveillance and defined as fever with positive blood smear. Asymptomatic parasitaemia was measured using active surveillance every 4 weeks and defined as molecular detection of malaria parasites from a DBS by LAMP. Measures of placental malaria at the time of delivery included: the detection of malaria parasites in placental blood by both microscopy and LAMP, and histopathologic evidence of placental malaria (parasites and/or pigment) from placental biopsies. Birth outcomes assessed included: LBW (< 2500 g), PTB (< 37 weeks gestational age), and SGA (birth weight < 10th percentile for gestational age according to East African fetal weight standards) [15]. In this study, an East African fetal weight standard was used because international growth standards (such as the Intergrowth-21st and the WHO growth curves) have shown greater variance of estimated fetal weight between countries, especially later in gestation [16, 17]. Utilizing the composite international standards above would have under- (Intergrowth-21st) or over-estimated (WHO) the incidence of SGA compared to the East African standards. There were eight cases of twin gestation; four were monochorionic–diamniotic and four were dichorionic–diamniotic. In these cases, outcomes were considered positive if present in at least one placenta and/or child.

### Statistical methods

Data were double entered into a Microsoft Access database. Data analysis was done using Stata 14 (Stata Corp, College Station TX). For baseline characteristics, comparison of proportions was done using the $\chi^{2}$ test and the one way anova test for normally-distributed continuous variables. Generalized linear Poisson regression models with robust standard errors were used to investigate associations between a categorical measure of malaria burden during pregnancy and measures of placental malaria as well as associations between a composite measure of malaria in pregnancy and adverse birth outcomes. Associations were expressed as relative risks. Multivariate analyses included adjustment for which drug was given for IPTp and gravidity. All p values were two-sided and values of < 0.05 were considered statistically significant.

### Ethical approval

Informed consent was obtained from all study participants. Ethical approval was obtained from the Uganda National Council of Science and Technology, the Makerere University School of Medicine Research and Ethics Committee, the Makerere University School of Biomedical Sciences Research and Ethics Committee, and the University of California, San Francisco, Committee on Human Research.

## Results

### Characteristics of malaria infection status during pregnancy

A total of 57/282 (20.2%) women had symptomatic malaria during pregnancy. Of these, 44/282 (15.6%) had only one episode of symptomatic malaria during pregnancy and 13/282 (4.6%) had more than one episode (2–3 episodes), resulting in a total of 72 malaria episodes. Asymptomatic parasitaemia during pregnancy was detected by LAMP on DBS in 230/282 (81.6%) of women; 162/282 (57.4%) had asymptomatic parasitaemia with < 50% of their monthly DBS samples positive for malaria parasites and 68/282 (24.1%) had ≥ 50% of their DBS samples positive for malaria parasites.

**Fig. 1:**
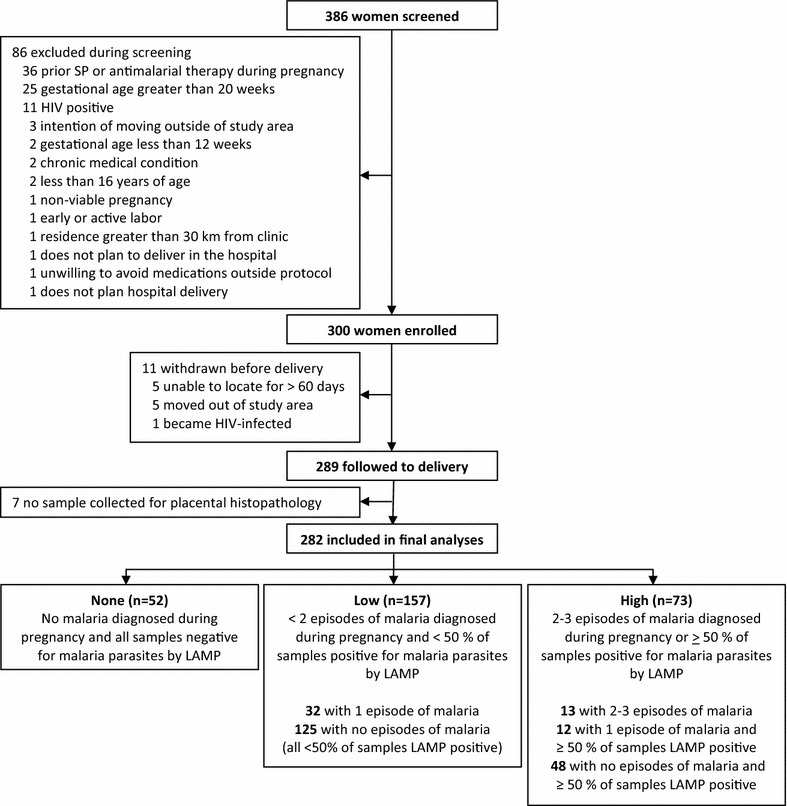
Flow of study participants

To investigate the associations between the frequency of malaria infection during pregnancy and placental malaria, a categorical variable of malaria burden during pregnancy was created. Three groups representing increasing malaria burden were defined as follows: (1) “none” = women who had no episodes of symptomatic malaria or asymptomatic parasitaemia during pregnancy; (2) “low” = women with 0–1 episode of symptomatic malaria and < 50% of DBS samples positive for parasites by LAMP during pregnancy; and (3) “high” = women who had ≥ 2 episodes of symptomatic malaria or ≥ 50% of their DBS samples positive for parasites by LAMP during pregnancy (Fig. 1). Based on these definitions, there were 52 (18.4%) women with no malaria infection detected during pregnancy, 157 (55.7%) women with low malaria burden in pregnancy, and 73 (25.5%) women with high malaria burden during their pregnancy.

Baseline characteristics of women stratified by malaria infection status during pregnancy are presented in Table 1. There were no differences in maternal age or gestational age at enrollment. Ownership of a long-lasting insecticide-treated bed net at enrollment was > 85% across all three groups with no statistically significant differences and all women were given an LLIN following enrollment. The proportion of primigravid women increased with higher malaria burden (21.2, 35.0, and 46.6% in the none, low, and high groups, respectively; p = 0.043). Wealth was inversely associated with malaria burden during pregnancy—the lowest tertile of wealth had the highest proportion of women with high malaria burden (39.7% vs. 32.9% and 27.4% of women in the middle and highest wealth tertiles, respectively, p = 0.026). Malaria burden during pregnancy was also associated with assigned IPTp regimens (p < 0.001), with women randomized to SP every 8 weeks having the highest burden of malaria and women randomized to DP every 4 weeks having the lowest burden of malaria.

**Table 1:** Characteristics of study participants by infection status during pregnancy

Characteristic	Malaria infection status during pregnancy			p value
	None (n = 52)	Low (n = 157)	High (n = 73)	
Age at enrollment in years, mean (SD)	23.5 (4.1)	22.1 (3.8)	21.0 (4.2)	0.989
Gestational age at enrollment in weeks, mean	15.6 (2.2)	15.2 (2.0)	15.5 (1.9)	0.857
Primigravida, n (%)	11 (21.2%)	55 (35.0%)	34 (46.6%)	0.043
Bed net ownership at enrollment, n (%)				
None	2 (3.9%)	20 (12.7%)	8 (11.0%)	
Untreated net	4 (7.7%)	2 (1.3%)	2 (2.7%)	0.071
LLIN	46 (88.5%)	135 (86.0%)	63 (86.3%)	
Household wealth index, n (%)				
Lowest tertile	13 (25.0%)	54 (34.4%)	29 (39.7%)	
Middle tertile	12 (23.1%)	57 (36.3%)	24 (32.9%)	0.026
Highest tertile	27 (51.9%)	46 (29.3%)	20 (27.4%)	
IPTp treatment arm, n (%)				
SP every 8 weeks	7 (13.5%)	40 (25.5%)	51 (69.9%)	
DP every 8 weeks	18 (34.6%)	53 (33.8%)	17 (23.3%)	< 0.001
DP every 4 weeks	27 (51.9%)	64 (40.8%)	5 (6.9%)	

### Associations between malaria infection status during pregnancy and measures of placental malaria

**Fig. 2:**
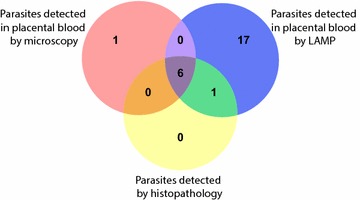
Distribution of parasites detected in placental samples based on method of detection

Three different methods of measuring placental malaria were evaluated: microscopy of placental blood smear, LAMP detection of parasite DNA in placental blood, and histopathologic detection of malaria infection (pigment or parasites) of placental biopsies. Risks of placental malaria were: 8/280 women (2.9%) by microscopy of placental blood smears, 24/280 women (8.6%) by LAMP of placental blood, and 105/282 women (37.2%) by placental histopathology. All 105 placentas positive by histopathology had pigment in fibrin indicative of past infection, but only 7 had parasites indicative of concomitant active infection. There were a total of 25 cases of placental malaria in which parasites were detected by any method (Fig. 2). Of the three methods, LAMP was the most sensitive in detecting malaria parasites, identifying 24 of 25 cases (96.0%); placental blood microscopy and histopathology each detected seven cases (7/25, 28.0%). Six cases were diagnosed by all three methods. One case was diagnosed by both histopathology and LAMP, but not placental blood smear. Finally, one case was detected only by placental blood smear, and not by LAMP or histopathology.

Associations between malaria burden during pregnancy, gravidity, and IPTp drug regimen with three different definitions of placental malaria were assessed. For analyses when placental malaria was defined as the detection of parasites in placental blood by microscopy or LAMP, the “none” and low malaria burden groups were combined to form the reference group due to small numbers. Univariate analysis showed that high malaria burden during pregnancy, primigravidity, and the use of SP for IPTp were all associated with an increased risk of placental malaria diagnosed by any method (Table 2). On multivariate analysis, evaluating the independent effects of malaria burden during pregnancy, gravidity, and IPTp drug, women with high malaria burden had a 14-fold higher risk of placental malaria by microscopy and a fourfold higher risk by LAMP, compared to women with none to low malaria burden (aRR 14.2, 95% CI 1.80–111.6, p = 0.01 and aRR 4.06, 95% CI 1.73–9.51, p = 0.001, respectively; Table 2). By histopathology, a clear dose-dependent effect was observed, whereby increasing malaria burden was associated with higher risks of placental malaria (aRR 3.27, 95% CI 1.32–8.12, p = 0.01 for low burden and aRR 7.07, 95% CI 2.84–17.6, p < 0.001 for high burden; Table 2). Primigravidity remained an independent risk factor for placental malaria by all methods on multivariate analysis (aRR 9.22, 3.05, and 2.89 for microscopy, LAMP, and histopathology, respectively; Table 2). IPTp with SP was also an independent risk factor for placental malaria by LAMP (aRR 3.86, 95% CI 1.56–9.56, p = 0.003), but not for placental malaria diagnosed by blood microscopy or histopathology. Findings were similar in a sensitivity analysis where women with symptomatic malaria during pregnancy were excluded, and associations between the proportion of samples with asymptomatic parasitaemia and various measures of placental malaria were evaluated (Additional file 1: Table S1).

**Table 2:** Associations between malaria infection status during pregnancy and measures of placental malaria

Risk factor	Category	Risk	Univariate		Multivariate	
			RR (95% CI)	p value	aRR (95% CI)	p value
Placental blood positive for malaria parasites by microscopy^a^ (n = 8)						
Malaria burden in pregnancy	None	0/52 (0%)	Reference group		Reference group	
	Low	1/156 (0.6%)				
	High	7/72 (9.7%)	20.2 (2.52–162.2)	0.005	14.2 (1.80–111.6)	0.01
Gravidity	Multigravida	1/182 (0.6%)	Reference group		Reference group	
	Primigravida	7/98 (7.1%)	13.0 (1.62–104.5)	0.02	9.22 (1.16–73.4)	0.04
IPTp drug	DP	3/184 (1.6%)	Reference group		Reference group	
	SP	5/96 (5.2%)	3.19 (0.78–13.1)	0.11	1.16 (0.32–4.15)	0.82
Placental blood positive for malaria parasites by LAMP^a^ (n = 24)						
Malaria burden in pregnancy	None	1/52 (1.9%)	Reference group		Reference group	
	Low	5/156 (3.2%)				
	High	18/72 (25.0%)	8.67 (3.57–21.0)	< 0.001	4.06 (1.73–9.51)	0.001
Gravidity	Multigravida	8/182 (4.4%)	Reference group		Reference group	
	Primigravida	16/98 (16.3%)	3.71 (1.65–8.38)	0.002	3.05 (1.41–6.62)	0.005
IPTp drug	DP	5/184 (2.7%)	Reference group		Reference group	
	SP	19/96 (19.8%)	7.28 (2.80–18.9)	< 0.001	3.86 (1.56–9.56)	0.003
Parasites or pigment on placental histopathology (n = 105)						
Malaria burden in pregnancy	None	4/52 (7.7%)	Reference group		Reference group	
	Low	47/157 (29.9%)	3.89 (1.47–10.3)	0.006	3.27 (1.32–8.12)	0.01
	High	54/73 (74.0%)	9.62 (3.71–24.9)	< 0.001	7.07 (2.84–17.6)	< 0.001
Gravidity	Multigravida	36/182 (19.8%)	Reference group		Reference group	
	Primigravida	69/100 (69.0%)	3.49 (2.53–4.81)	< 0.001	2.89 (2.12–3.94)	< 0.001
IPTp drug	DP	56/184 (30.4%)	Reference group		Reference group	
	SP	49/98 (50.0%)	1.64 (1.22–2.21)	0.001	1.02 (0.79–1.32)	0.87

^a^Results missing for 2 of the 282 participants

### Associations between a composite indicator of malaria in pregnancy and adverse birth outcomes

A total of 38 (13.5%) women had LBW infants, 26 (9.2%) women delivered preterm, and 57 (20.2%) had infants that were SGA. A composite indicator of gestational malaria burden and placental malaria was generated to explore associations between malaria in pregnancy with birth weight, and adverse birth outcomes. Women were grouped into the following categories: (1) “none” category with no symptomatic or asymptomatic malaria and no placental malaria, (2) low malaria burden and no placental malaria, (3) high malaria burden and no placental malaria, (4) any malaria burden (none, low, and high combined) and placental malaria with pigment only on histopathology, and (5) any malaria burden and placental parasites detected (by microscopy, LAMP, or histopathology) (Table 3). There were no differences in mean birth weights between categories 1–4. However, if parasites were detected in the placenta (category 5) mean birth weight was significantly lower (2598 vs. 2934, p = 0.02) and (2598 vs. 2962, p = 0.0003) compared to category 1 or when parasites were not detected in the placenta (categories 1–4) respectively. Furthermore, associations between this composite indicator of malaria in pregnancy, gravidity, and IPTp drug with adverse birth outcomes were investigated.

**Table 3:** Associations between a composite indicator of malaria in pregnancy and adverse birth outcomes

Risk factor	Category			Low birth weight (< 2500 gm)			Preterm birth (< 37 weeks)			Small for gestational age (< 10‰)		
		Malaria burden during pregnancy	Placental malaria	Risk	aRR^a^ (95% CI)	*p*	Risk	aRR^a^ (95% CI)	*p*	Risk	aRR^a^ (95% CI)	*p*
Composite indicator of malaria in pregnancy	1	None	None	6/47 (12.8%)	Reference		2/47 (4.3%)	Reference		9/47 (19.2%)	Reference	
	2	Low	None	8/110 (7.3%)	0.56 (0.20–1.55)	0.27	5/110 (4.6%)	1.06 (0.22–5.08)	0.95	16/110 (14.6%)	0.74 (0.35–1.58)	0.44
	3	High	None	3/18 (16.7%)	1.41 (0.36–5.54)	0.63	2/18 (11.1%)	3.33 (0.51–21.6)	0.21	4/18 (22.2%)	1.16 (0.40–3.36)	0.79
	4	Any	Pigment only^b^	10/82 (12.2%)	0.77 (0.28–2.13)	0.61	9/82 (11.0%)	1.74 (0.34–8.96)	0.51	17/82 (20.7%)	0.90 (0.42–1.92)	0.78
	5	Any	Parasites detected^c^	11/25 (44.0%)	2.85 (0.92–8.90)	0.07	8/25 (32.0%)	5.88 (1.02–34.0)	0.048	11/25 (44.0%)	1.85 (0.79–4.36)	0.16
Gravidity	Multigravida			18/182 (9.9%)	Reference		9/182 (5.0%)	Reference		30/182 (16.5%)	Reference	
	Primigravida			20/100 (20.0%)	1.60 (0.80–3.23)	0.19	17/100 (17.0%)	2.42 (0.84–7.00)	0.10	27/100 (27.0%)	1.43 (0.86–2.39)	0.17
IPTp drug	DP			22/184 (12.0%)	Reference		16/184 (8.7%)	Reference		34/184 (18.5%)	Reference	
	SP			16/98 (16.3%)	0.89 (0.44–1.83)	0.76	10/98 (10.2%)	0.67 (0.27–1.66)	0.39	23/98 (23.5%)	1.04 (0.63–1.71)	0.89

^a^Adjusted relative risk

^b^By histopathology

^c^By placental blood smear, placental LAMP, or histopathology

Compared to category 1 (“none” with no evidence of placental malaria), there was a non-significant trend towards increased risk of LBW, PTB, and SGA in category 3 (high malaria burden/no placental malaria) in univariate analysis (Additional file 1: Table S2). If parasites were detected in the placenta (Category 5), there was a significant association with LBW (RR 3.45, 95% CI 1.44–8.23, p = 0.005), PTB (RR 7.52, 95% CI 1.72–32.8, p = 0.007), and SGA (RR 2.30, 95% CI 1.10–4.80, p = 0.03) when compared to category 1 (Additional file 1: Table S2). There was no association with any of the adverse birth outcomes when categories 2 (low malaria burden with no placental malaria) and 4 (placental malaria with pigment only) were compared with category 1 (Additional file 1: Table S2). After adjustment for gravidity and IPTp, there still was a non-significant trend towards increased risk of LBW, PTB, and SGA in category 3 compared to category 1 (Table 3). Amongst women with parasites in the placenta, there still was a significant association with PTB (aRR 5.88, 95% CI 1.02–34.0, p = 0.048) compared to category 1. However, the associations with LBW and SGA within this category were not statistically significant although there were trends for increased risk for LBW and SGA (Table 3). Primigravidity was associated with non-significant trends for an increased risk for all 3 adverse birth outcomes and IPTp drugs were not associated with any of the adverse birth outcomes.

## Discussion

Associations between measures of symptomatic malaria and asymptomatic parasitaemia during pregnancy, placental malaria, and adverse birth outcomes were assessed in a well described cohort of Ugandan women enrolled in a clinical trial of IPTp. The burden of malaria was high in this cohort with asymptomatic parasitaemia detected in over 80% of women and over 20% of women having at least one episode of symptomatic malaria. Higher malaria burden during pregnancy was strongly associated with three different measures of placental malaria: the detection of malaria parasites in placental blood by microscopy or LAMP, and histopathologic evidence of placental malaria (parasites and/or pigment) from placental biopsies. When evaluating associations between a composite indicator of malaria in pregnancy and adverse birth outcomes (LBW, PTB, and SGA), the detection of malaria parasites in the placenta was found to be a statistically significant risk factor for PTB. However, it should be noted that due to the relatively small sample size of some categories of malaria in pregnancy, the lack of statistically significant associations with adverse birth outcomes may have been due to a lack of statistical power.

The results presented here are consistent with prior studies that demonstrated an association between peripheral parasitaemia during pregnancy and placental malaria [8, 10, 18]. De Beaudrap et al. showed that peripheral parasitaemia at enrollment (detected by rapid diagnostic test and microscopy) and the number of symptomatic malaria episodes were most strongly associated with placental malaria [8]. Cohee et al. similarly found that each episode of submicroscopic infection increased the risk of placental malaria by nearly fivefold [10]. Similarly, women with a high malaria burden during pregnancy had a much higher risk of placental malaria defined by microscopy, LAMP, or histopathology compared to women with no infections with malaria parasites detected or low malaria burden during pregnancy after adjusting for IPTp treatment arm and gravidity.

Prior longitudinal studies have also shown associations between peripheral *Plasmodium falciparum* infection during pregnancy and adverse pregnancy outcomes, such as LBW, PTB, and SGA [7, 8, 18, 19]. These studies have consistently found that increasing episodes of malaria infection in pregnancy (both submicroscopic and microscopic) are associated with higher risks of decreased birth weight. This study builds upon these findings by examining the relationship between both symptomatic malaria and asymptomatic parasitaemia and the intermediary effect of placental infection on adverse outcomes.

Associations between the presence of placental malaria and an increased risk of LBW and/or PTB have been previously reported in several cross-sectional studies [2, 3, 20, 21]. In this study a composite indicator was generated which included both measures of malaria burden during pregnancy and the presence of malaria pigment or parasites in placental samples. When evaluating the associations between this composite indicator and adverse birth outcomes, the detection of malaria parasites in placental samples was significantly associated with PTB (aRR = 5.88) in multivariate analyses and there was a non-significant trend towards increased risk for LBW and SGA with relative risks of considerable magnitude.

Low birth weight can be due to prematurity, or SGA (birth weight below the 10th percentile expected for the population at the given gestational age, usually from fetal growth restriction), or both. SGA infants have been shown to have twice the mortality risk compared to appropriate for gestational age infants in low and middle income countries, for both preterm and term births [22, 23]. Furthermore, SGA infants may have an increased risk of long-term neuro-cognitive impairment, even in term infants [24]. However, studies examining the relative contributions of LBW, SGA, and PTB in malaria in pregnancy have been sparse [8, 21, 25, 26], as accurate determination of gestational age by early ultrasound is frequently lacking.

As ultrasound technology has become more widely available in malaria-endemic regions, the importance of distinguishing between these factors has become more apparent. For example, Rijken et al. showed that in a Thai population, even treated malaria in pregnancy was associated with an increased risk of LBW, PTB, and SGA; SGA was present in 5% of malaria-affected pregnancies in non-LBW babies born after 39-weeks of gestation [27]. Thus, by not considering birth weight adjusted for gestational age, the true impact of malaria on pregnancy outcomes is underestimated, particularly in term pregnancies. Using a growth curve based on an East African population [15] in this study, the rate of SGA infants was 20.2%, while the rate of LBW and PTB were 13.5 and 9.2% respectively. The presence of parasites in the placenta was associated with a 1.8-fold increased risk of SGA in multivariate analysis. Although the detection of parasites in the placenta was significantly associated with only the adverse birth outcome of preterm delivery, caution should be taken in excluding the possibility that presence of placental parasites is associated with other adverse birth outcomes such as LBW or that other measures of malaria in pregnancy are associated with adverse birth outcomes. For example, women with a high burden of malaria during pregnancy but no evidence of placental malaria (category 3) had non-significant trends towards an increased risk of adverse birth outcomes and the precision of these estimates were limited by a small sample size (n = 18).

Primigravidity is a well-established risk factor for increased susceptibility to malaria in pregnancy and adverse clinical outcomes [5, 28], consistent with findings from this study. Primigravidas had a higher frequency of malaria in pregnancy compared to multigravidas, were more likely to experience higher malaria burden, and had a higher risk of placental malaria at the time of delivery. There was also a trend towards increased risk of adverse birth outcomes (LBW, PTB, and SGA) in primigravidas, but these associations did not reach statistical significance after controlling for a composite indicator of malaria in pregnancy probably because of adjustment for malaria during pregnancy, which is on the causal pathway between gravidity and birth outcome. The gravidity-dependent differences in susceptibility to malaria during pregnancy, as well as the risk differences of clinical sequelae, is thought to be related to the development of immunity specific to placental malaria in second and subsequent pregnancies [29, 30].

Of note, in this study LAMP was used to screen for asymptomatic parasitaemia. LAMP is more sensitive than microscopy, in both asymptomatic and symptomatic cases in pregnancy [9, 31], and so the detection threshold is likely lower than previous studies that relied primarily on microscopy to detect malaria parasites [8, 18]. Furthermore, this study also investigated the impact of submicroscopic infection in the placenta. Importantly, even though this cohort was part of a randomized clinical trial of different IPTp regimens, the women had high rates of both asymptomatic parasitaemia and symptomatic malaria episodes, especially in the IPTp arm with the current WHO standard of three doses of SP as previously reported [12]. This highlights the need to improve current strategies for prevention of malaria in pregnancy, and adverse outcomes.

Three methods of detection of placental malaria were directly compared: placental blood smear, LAMP of placental blood, and histopathology. While a high prevalence (37%) of placental malaria with only pigment on histopathology was observed, this measure was not significantly associated with adverse birth outcomes. This is not surprising given that pigment in the placenta represents prior infection. In contrast, the presence of parasites in the placenta, diagnosed by any of the three methods, was significantly associated with PTB and with an increased risk for LBW and SGA. LAMP was the most sensitive of the three, demonstrating that this method may provide the optimal diagnostic test for placental malaria. Additionally, LAMP has clear advantages over the other two methods in low resource settings—it is easy to learn, does not require specialized equipment, and is low cost with rapid results. In contrast, placental blood smear is less sensitive (detecting 1/3 of the cases diagnosed by LAMP in the study cohort), and placental histopathology is resource-intensive and not routinely available in malaria-endemic settings.

This study did have limitations. Malaria burden prior to enrollment at 12–20 weeks gestation was not evaluated, which may have influenced both measures of placental malaria and birth outcomes. Other factors that could affect fetal growth, birth weight, and prematurity were not investigated, such as maternal nutritional status, child spacing, maternal anaemia, genetics, other infections, and smoking. Serial ultrasound examinations during the pregnancy were not performed, which could help to distinguish constitutionally SGA (for example from genetic factors) from SGA due to fetal growth restriction. Given the relatively small sample size and low frequency of several of the variables of interest, the lack of statistically significant associations may have been due to insufficient power rather than true negative findings (type II error). However, this study also has several strengths. One is the longitudinal design with frequent (monthly) measurements of parasitaemia by molecular methods, obstetric dating of gestational age by ultrasound prior to 20 weeks gestation, and comprehensive analysis of placental malaria.

## Conclusions

Increasing malaria burden in pregnancy was associated with primigravidity, lower household wealth, and IPTp treatment regimen (especially treatment with SP). Higher malaria burden in pregnancy was associated with placental malaria, and LAMP was the most sensitive measure of malaria parasites in the placenta compared with placental blood microscopy and histopathology. The detection of malaria parasites in placental samples was associated with an increased risk of adverse birth outcomes.

## Additional file

**Additional file 1.** Additional tables.
