# Supplementary material for: Relationships between infection with Plasmodium falciparum during pregnancy, measures of placental malaria, and adverse birth outcomes
Source: Malar J. 2017 Oct 5;16:400. doi: 10.1186/s12936-017-2040-4 (PMC5629777; doi:10.1186/s12936-017-2040-4)
Supplement: Supplementary file 1 — Additional file 1. Additional tables. [file 12936_2017_2040_MOESM1_ESM.docx]

**Table S1.** Associations between asymptomatic parasitemia during pregnancy and measures of placental malaria after excluding women with any symptomatic malaria during pregnancy

| **Risk factor** | **Category** | | **Risk** | **Univariate** | | **Multivariate** | |
| --- | --- | --- | --- | --- | --- | --- | --- |
|  |  |  |  | **RR (95% CI)** | **p-value** | **aRR (95% CI)** | **p-value** |
| **Placental blood positive for malaria parasites by microscopy^a^ (n=3)** | | | | | | | |
| Proportion of samples LAMP positive | | 0% | 0/52 (0%) | reference group | | reference group | |
|  |  | < 50% | 0/125 (0%) |  |  |  |  |
|  |  | > 50% | 3/47 (6.4%) | N/A | 0.001 | N/A | <0.001 |
| Gravidity | | Multigravida | 0/149 (0%) | reference group | | reference group | |
|  |  | Primigravida | 3/75 (4.0%) | N/A | 0.01 | N/A | <0.001 |
| IPTp drug | | DP | 1/159 (0.6%) | reference group | | reference group | |
|  |  | SP | 2/65 (3.1%) | 4.89(0.45-53.3) | 0.19 | 1.64(0.17-15.3) | 0.67 |
| **Placental blood positive for malaria parasites by LAMP^a^ (n=14)** | | | | | | | |
| Proportion of samples LAMP positive | | 0% | 1/52 (1.9%) | reference group | | reference group | |
|  |  | < 50% | 3/125 (2.4%) |  |  |  |  |
|  |  | > 50% | 10/47 (21.3%) | 9.41(3.08-28.8) | <0.001 | 4.69(1.83-12.1) | 0.001 |
| Gravidity | | Multigravida | 6/149 (4.0%) | reference group | | reference group | |
|  |  | Primigravida | 8/75 (10.7%) | 2.65(0.96-7.37) | 0.06 | 2.24(0.88-5.70) | 0.09 |
| IPTp drug | | DP | 3/159 (1.9%) | reference group | | reference group | |
|  |  | SP | 11/65 (16.9%) | 8.97(2.58-31.2) | 0.001 | 5.01(1.70-14.8) | 0.004 |
| **Parasites or pigment on placental histopathology (n=72)** | | | | | | | |
| Proportion of samples LAMP positive | | 0% | 4/52 (7.7%) | reference group | | reference group | |
|  |  | < 50% | 36/125 (28.8%) | 3.74 (1.40-10.0) | 0.008 | 3.05 (1.24-7.51) | 0.02 |
|  |  | > 50% | 32/48 (66.7%) | 8.67 (3.30-22.7) | <0.001 | 6.56 (2.65-16.3) | <0.001 |
| Gravidity | | Multigravida | 22/149 (14.8%) | reference group | | reference group | |
|  |  | Primigravida | 50/76 (65.8%) | 4.46 (2.93-6.78) | <0.001 | 3.74 (2.47-5.65) | <0.001 |
| IPTp drug | | DP | 46/159 (28.9%) | reference group | | reference group | |
|  |  | SP | 26/66 (39.4%) | 1.36 (0.92-2.00) | 0.12 | 0.89 (0.64-1.24) | 0.50 |

^a^Results missing for 1 of the 225 participants

**Table S2.** Unadjusted associations between a composite indicator of malaria in pregnancy and adverse birth outcomes

| **Risk factor** | **Category** | | | **Low Birth Weight (< 2500 gm)** | | | **Preterm Birth (<37 weeks)** | | | **Small for Gestational Age (<10%ile)** | | |
| --- | --- | --- | --- | --- | --- | --- | --- | --- | --- | --- | --- | --- |
|  |  | **Malaria burden during pregnancy** | **Placental malaria** | **Risk** | **RR (95% CI)** | ***p*** | **Risk** | **RR (95% CI)** | ***p*** | **Risk** | **RR (95% CI)** | ***p*** |
| Composite indicator of malaria in pregnancy | 1 | None | None | 6/47 (12.8%) | reference | | 2/47 (4.3%) | reference | | 9/47 (19.2%) | reference | |
|  | 2 | Low | None | 8/110 (7.3%) | 0.57 (0.21-1.55) | 0.27 | 5/110 (4.6%) | 1.07 (0.21-5.33) | 0.94 | 16/110 (14.6%) | 0.76 (0.36-1.60) | 0.47 |
|  | 3 | High | None | 3/18 (16.7%) | 1.31 (0.36-4.68) | 0.68 | 2/18 (11.1%) | 2.61 (0.40-17.2) | 0.32 | 4/18 (22.2%) | 1.16 (0.41-3.31) | 0.78 |
|  | 4 | Any | Pigment only**^a^** | 10/82 (12.2%) | 0.96 (0.37-2.47) | 0.93 | 9/82 (11.0%) | 2.58 (0.58-11.5) | 0.21 | 17/82 (20.7%) | 1.08 (0.52-2.24) | 0.83 |
|  | 5 | Any | Parasites detected**^b^** | 11/25 (44.0%) | 3.45 (1.44-8.23) | 0.005 | 8/25 (32.0%) | 7.52 (1.72-32.8) | 0.007 | 11/25 (44.0%) | 2.30 (1.10-4.80) | 0.03 |
| Gravidity | Multigravida | | | 18/182 (9.9%) | reference | | 9/182 (5.0%) | reference | | 30/182 (16.5%) | reference | |
|  | Primigravida | | | 20/100 (20.0%) | 2.11 (1.18-3.77) | 0.01 | 17/100 (17.0%) | 3.61 (1.68-7.76) | 0.001 | 20/100 (20.0%) | 2.11 (1.18-3.77) | 0.01 |
| IPTp drug | DP | | | 22/184 (12.0%) | reference | | 16/184 (8.7%) | reference | | 34/184 (18.5%) | reference | |
|  | SP | | | 16/98 (16.3%) | 1.42 (0.79-2.55) | 0.24 | 10/98 (10.2%) | 1.26 (0.61-2.62) | 0.53 | 16/98 (16.3%) | 1.42 (0.79-2.55) | 0.24 |

**^a^** by histopathology

**^b^** by placental blood smear, placental LAMP, or histopathology
